# Salt restriction induced hyponatremia in hypertensive patients in Rwanda: A case control study

**DOI:** 10.1371/journal.pone.0308457

**Published:** 2024-12-03

**Authors:** Emmanuel BIZIMANA, Eric RUTAGANDA, Adeline MUGENI, Prisca UWUMURYANGO

**Affiliations:** 1 Department of Internal Medicine, College of Medicine and Health Sciences, University of Rwanda, Kigali, Rwanda; 2 Department of Internal Medicine, Kigali University Teaching Hospital, University of Rwanda, Kigali, Rwanda; 3 Department of Internal Medicine, Rwanda Military Hospital, University of Rwanda, Kigali, Rwanda; Barking Havering and Redbridge Hospitals NHS Trust: Barking Havering and Redbridge University Hospitals NHS Trust, UNITED KINGDOM OF GREAT BRITAIN AND NORTHERN IRELAND

## Abstract

**Background:**

Salt restriction is a fundamental principle in the non-pharmacological management of hypertension. The World Health Organization recommends a daily sodium intake of less than 2 g/day. In East African countries, particularly Rwanda, there is a known prevalence of low sodium intake, with a mean sodium intake of 1.6 g/day. However, despite this dietary habit, the national protocol for treating hypertension, as well as common clinical practice, often fail to account for the low salt intake in Rwandan communities. Hypertensive patients are still frequently advised to reduce their salt intake, and in some cases, they are instructed to eliminate salt entirely.

**Objectives:**

This study was designed to determine the association between salt restriction and hyponatremia in hypertensive patients.

**Methods:**

A case-control study was conducted over a period of 6 months at two tertiary hospitals in Rwanda, with hyponatremia as the outcome variable and salt restriction as the exposure variable. Age, gender, and use of diuretics were matched between the case and control groups. Serum sodium concentrations were measured, and participants were then categorized into groups. Questionnaires were used for interviews.

**Results:**

245 participants meeting the inclusion criteria were selected, with 110 (44.9%) classified as cases and 135 (55.1%) as controls. Among them, 159 (64.8%) participants were restricted from consuming salt, with 74 (46.5%) following a salt-free diet. The odds of developing hyponatremia were 9.90 (95% CI, p < 0.001) among salt-restricted participants.

**Conclusion:**

There is a strong association between salt restriction and hyponatremia in hypertensive patients on treatment in this study.

## Introduction

Salt intake is essential to life [1]. Despite the physiological need of salt, when taken in excess, it is linked to raised blood pressure; hence many guidelines for treatment of hypertension recommend salt restriction to control blood pressure [2]. WHO recommends a sodium intake of 2 g per day [3].

Rwanda, as any other east Africa country, has a low sodium diet intake with an average of 1.6 g per day per person [4]. Despite this low salt intake, national protocol for treatment of hypertension still recommends a salt restriction [5]. Additionally, like in many developing countries, Rwanda population has a low protein intake. When combined, low salt intake and low protein intake can lead to low solute intake, a condition known to cause hyponatremia by decreasing free water excretion capacity [6].

Hyponatremia, especially in elderly has many adverse outcomes including poor cognitive function, and some studies suggested that low salt intake might be linked with increased mortality, similar to high salt intake [7].

As stated above, with average sodium intake of 1.6 g/day, most of Rwandans’ daily sodium consumption falls below the WHO-recommended intake of 2 g/day; despite this, many hypertensive patients who are on treatment are still advised to restrict their salt intake further, and some are even prescribed the salt-free diets. This, combined with other factors of low solute diet (such as low protein) increases the risk of developing hyponatremia.

There is currently no available data in Rwanda on effect of this recommendation of salt restriction to serum sodium concentration. We hypothesize that salt restriction in Rwanda is highly associated with hyponatremia; we aim to determine any association between salt restriction and hyponatremia in hypertensive patients undergoing treatment.

## Methods

We conducted a case control study at Kigali University teaching hospital (CHUK) and Rwanda Military Hospital (RMH) in Kigali, Rwanda over a period of 6 months from 26^th^ August 2019 to 26^th^ February 2020. The Study population was the adult hypertensive patients on treatment; we included the hypertensive patients above 18 years of age who provided consent and had been receiving treatment for hypertension for at least 3 months. We excluded those who didn’t sign consent form, those who were in fluid overload and patients on hypertensive treatment below 3 months.

The Case group comprised of hypertensive patients on treatment who had hyponatremia, while the control group consisted of hypertensive patients on treatment who did not have hyponatremia. The exposure was salt restriction. To minimize the confounders, we matched cases and controls on age, gender, use of diuretics and duration of the treatment.

All participants who met the inclusion criteria were taken blood sample for serum sodium concentration analysis and then grouped into either case or control group with respect to matching criteria. Participants were interviewed using a questionnaire to gather information on their dietary salt intake, past medical history and drugs history.

Sample size consisted of the hypertensive patients who presented at CHUK and RMH during the study period. Collected data was entered, and analyzed using Epi-info version7.2 and stata14 software. Logistic regression was used to calculate odd ratio at 95%CI for different variables for their association with hyponatremia in our study population and chi-square test used for p-value. Test results were considered statistically significant if the chi-square test p-value was < 0.05. Participants’ data were retained confidentially and stored anonymously. Written informed consent was signed by participants or their care takers.

The ethical approval to carry out the study was given by the University of Rwanda Institutional Review Board (IRB) No 199/CMHS IRB/2019 as well as the ethics committee of University Teaching Hospital of Kigali, referenced as Ref: EC/CHUK/167/2019; and Rwanda Military Hospital, referenced as Ref: RMH IRB/044/2019.

## Results

A sample of 245 participants meeting inclusions criteria was taken. Cases (hypertensive patients with hyponatremia) were 110 (44.9%) and controls were 135 (55.1%). All participants were retrospectively assessed for the exposure to salt restriction versus no salt restriction. The cases and controls were matched by age, gender, use of diuretics and duration on treatment of hypertension.

The mean age was 63.25 ± 13.5 in case and 61.4 ±13.9 in controls. Males were 39 (46.6%) in case group versus 45 (54.4%) in control group while Females were 71 (44.1%) in case group versus 90 (55.9%) in control group. 74 Participants were using diuretics as the antihypertensive medications, of whom 42 (56.7%) were in control group and 32 (43.3%) were in case group.

The mean serum sodium concentration in cases was 130.57 ± 3.87 meq/L and 138. ± 2.14 meq/L in controls. Overall prevalence of hypertensives on salt restriction (decreased salt intake compared to family salt diet) was 159 (64.8%), of which 74 (46.5%) were taking salt free diet. Among cases (n = 110), 76 (69%) had mild hyponatremia, 28 (25%) had moderate hyponatremia and 6 (5%) had severe hyponatremia.

Among cases, 98 (89.1%) were exposed (salt restricted), while among controls, 61 (45.1%) were exposed. The odds ratio of having hyponatremia if being exposed (salt restricted) was 9.90 (P-value<0.001) as shown in Table 1.

**Table 1:** Association of hyponatremia with salt restriction in hypertensive patients.

			sNa conc.(outcome)				p-value	OR
			CASES		CONTROLS			
			N	(%)	N	(%)		
**EXPOSURE**	*Salt restricted*	**YES**	98	(89.1)	61	(45.1)	0.000	**9.907**
		**NO**	12	(10.9)	74	(54.9)		

Further analysis of other risk factors and comorbidities revealed a potential association between heart disease and hyponatremia with an odds ratio of 3.00; However, this association was not statistically significant, with a p-value of 0.060. The same trend was observed for renal disease, with an odds ratio of 2.33 and a p-value of 0.090 as shown in Table 2.

**Table 2:** Factors associated with hyponatremia in hypertensive patients on treatment.

	N	CASES	CONTROLS	P-value	OR
		%	%		
**Drug used**					
Thiazide	45	35.6%	64.4%	0.208	1.652
CCB	167	41.9%	58.1%	0.346	0.776
ACEI	29	48.3%	51.7%	0.622	1.015
ARB	100	48.0%	52.0%	0.298	1.110
B Blockers	40	52.5%	47.5%	0.237	1.003
Loop Diuretics	29	55.2%	44.8%	0.197	1.663
**Comorbidities**					
Heart Disease	13	69.2%	30.8%	0.060	3.030
Renal Disease	42	50.0%	50.0%	0.090	2.337
Diabetes mellitus	89	44.9%	55.1%	0.823	1.061

## Discussion

Salt restriction to 5 g/day (2g/day of sodium) is recommended by WHO as one way of treating hypertension [3], and national sodium intake average in Rwanda is reported to be 1.6g/day [4]. In our study, serum sodium concentration of 245 participants who were on antihypertensive drugs has been analyzed with 110 (44.9%) of Cases (participants with hypertension on treatment who developed hyponatremia) and 135 (55.1%) of Controls.

The analysis of study findings revealed a strong association between hyponatremia and salt restriction in hypertensive patients with an odds ratio of 9.9, P-value <0.005.

This association supports the findings of Angela J. D and Mark I. M., who postulated in their systematic review titled: “The Hyponatremia epidemic: A Frontier Too Far? Reviewing the current evidence of low salt diet and prevalence of hyponatremia” that the increase of prevalence of hyponatremia is linked to the widespread adoption of low salt diet policy [8].

Drake-Holland AJ and Noble MIM postulated that low salt intake can lead to hyponatremia [9]. The same association between salt restriction and hyponatremia also has been demonstrated by Giordano M. et al in their study on seasonal variation of serum sodium in the emergency department which showed that hyponatremia during summer is partly linked to decreased salt intake during that period [10].

The above association is explained by the mechanism of hyponatremia resulting from low solute intake; in this category of hyponatremia which does not dependent on ADH, low solute intake (low salt intake and low protein intake) leads to decreased free water excretion capacity leading to hyponatremia [6, 11]. This low solute intake is especially seen in our population: low sodium intake as observed by Powles J. et al in their study entitled “Global, regional and national sodium intakes in 1990 and 2010: a systematic analysis of 24 h urinary sodium excretion and dietary surveys worldwide” [4] and low protein intake reported in the study of Rawlins R. et al evaluating the impact of livestock donation programs in Rwanda on nutritional outcomes [12]

The diuretics were not found to be independent risk factors for hyponatremia; loop diuretics had an odds ratio of 1.663 with a p-value > 0.05, the odds of getting hyponatremia when using thiazide diuretics was 1.652 with a p-value > 0.05. Same findings were found by Almas, A. et al in their study which showed that the association between use of diuretics and hyponatremia in hypertensive adult patients is not significant [13].

In this study, heart disease and renal disease were not independent risk factors for developing hyponatremia; same findings were observed by Almas, A. et al [13].

The strength of this study is a two sites study with a case control design; it is the first study done to assess association of salt restriction and hyponatremia in Rwanda. However, there are some limitations including lack of urinary sodium concentration measurements and inability to achieve the initially planned sample size ratio of 1:2 (case: control) due to study period constraints.

## Conclusion

Salt restriction for hypertensive patients on treatments in Rwanda is associated with hyponatremia. This association can be explained by two combined mechanisms: low salt intake in Rwanda (mean sodium intake in Rwanda is 1.6g/d) and low protein intake (developing country); and both lead to decreased free water excretion capacity resulting in hyponatremia.

We recommend to develop a local salt intake guideline that takes into accounts the aforementioned mechanisms. Furthermore, additional studies are needed to thoroughly assess this association.

## Supporting information

S1 Data set (SAV)

## Abbreviations

ACEI: Angiotensin-Converting Enzyme Inhibitors
ADH: Antidiuretic Hormone
AHA/ACC: American Heart association/American College of Cardiology
ARB: Angiotensin Receptor Blocker
BP: Blood Pressure
CCB: Calcium Channel Blocker
CHUK: Centre Hospitalier Universitaire de Kigali
CMHS: College of Medicine and Health Sciences
DM: Diabetes mellitus
IRB: Institutional Review Board
MOH: Ministry of Health
Na: Sodium
NaCl: Sodium chloride
OR: Odd ratio
RMH: Rwanda Military Hospital
sNa: Serum sodium concentration
UR: University of Rwanda
WHO: World Health Organization
